# The Impact of Parental Remote Migration and Parent-Child Relation Types on the Psychological Resilience of Rural Left-Behind Children in China

**DOI:** 10.3390/ijerph17155388

**Published:** 2020-07-27

**Authors:** Hongsheng Liu, Lige Liu, Xiaoyi Jin

**Affiliations:** 1 School of Public Administration, Xi’an University of Architecture and Technology, Xi’an 710055, China; hongshengliu@xauat.edu.cn; 2 College of Humanities & Social Development, Northwest Agriculture and Forestry University, Yangling 712100, China; ligeliu@nwsuaf.edu.cn; 3 School of Public Policy and Administration, Xi’an Jiaotong University, Xi’an 710049, China

**Keywords:** parental remote migration, parent-child relation types, psychological resilience, rural left-behind children

## Abstract

Using survey data of middle school students from Ye county in Henan province and Chenggu and Ningqiang county in Shaanxi province, China, adopting latent class analysis and hierarchical linear regression, this study analyzes the impact of parental remote migration and parent-child relation types on the psychological resilience of rural left-behind children. The results show that: Only mother’s remote migration has a significantly negative impact on the psychological resilience of rural left-behind children, the time of parental first migration, the distance of father’s migration, and children’s migration have no significant impacts; parent-child relation of “alienation connection and weak function” or parent-child relation combination of “parental alienation connection and weak function” is the most unfavorable factor for the psychological resilience of rural left-behind children, while father-child relation of “close connection but lacking function”, mother-child relation of “intimate connection and strong function”, and combination of “paternal close connection but lacking function - maternal intimate connection and strong function” are the most favorable factors. There is gender difference in the impact of father-child relation types and mother-child relation types, the impact of father-child relation types is stronger than that of mother-child relation types; harmonious parental relation, supportive friends, caring teachers, and moderate home-school interaction are favorable for the psychological resilience of rural left-behind children.

## 1. Introduction

With rapid urbanization in China, the scale of migrant workers has been increasing. It was estimated that China’s migrant workers might reach 307 million in 2025 and 327 million in 2030 [1]. Large-scale population migration has become a social phenomenon worthy of attention. Restricted by institutional and economic factors, it is hard for migrant workers to bring their children to cities where they work, which creates a large group of left-behind children in rural China. According to China’s Ministry of Civil Affairs, left-behind children are minors whose both parents have gone to cities for work, or one parent has gone to cities for work while the other has no guardianship ability, and in 2018, there were still 6.97 million left-behind children in rural China [2].

It has been recognized that rural left-behind children are disadvantaged in many aspects. After parents migrated to cities, family members reduced, their roles changed, left-behind children had to separate from parents, and they could not interact with parents like before, which might affect parent-child relation and family function. Especially, parental remote migration, damaged family structure, and weakened family function may force left-behind children to be trapped in unfavorable situation and threaten their mental health.

Numerous studies have confirmed that parental migration significantly affects left-behind children’s mental health. Left-behind children report more mental health problems than their peers [3]. They score higher in negative mental health indicators (e.g., depression and anxiety), but score lower in positive mental health indicators (e.g., self-esteem and happiness) [4]. Meanwhile, studies have also revealed that some left-behind children do not encounter mental health problems [5]. There is no significant difference in happiness, security, self-esteem, and life satisfaction between left-behind children and their peers [6,7]. Some left-behind children’s performance in health, nutrition, and education is not worse than, or even better than, their peers [8], which indicates that being left-behind does not definitely lead to mental health problems among left-behind children.

Some children experiencing adversities can also survive, adapt, and develop normally [9,10]. Positive psychology uses “resilience” to explain this phenomenon of “adaptation to adversities”. There are many definitions about resilience. Some define it as a kind of capability [11,12], others define it as a kind of status or result [13,14], still others define it as a process [15,16], and most emphasize the value of resilience for children’s development. Resilience is regarded as a regulator that modulates the negative effect of adversities on children’s development [10], or a buffer that plays a positive role when children face negative events [17], it can minimize the psychological and physiological costs of children when coping with adversities [18]. It is shown that children with high resilience can not only overcome adversities, but also develop better [19]. Therefore, resilience has positive and practical significance for the development of left-behind children.

Related studies have focused on exploring the relation between resilience and children’s mental health, and found that resilience was positively correlated with mental health [20], it could not only negatively affect negative mental health indicators (e.g., anxiety and withdrawal) [21,22], but also positively affect positive mental health indicators (e.g., happiness and satisfaction) [23,24]. Thus, it can be seen that resilience can guarantee and promote children’s mental health, and it is of great value to study the influencing factors of left-behind children’s resilience.

However, few studies have analyzed the influencing factors of left-behind children’s resilience. Especially, parental migration is a major event in left-behind children’s life course, its spatial and temporal characteristics may affect left-behind children’s resilience, which deserves more attention. Meanwhile, parent-child separation threatens parent-child relation, but parent-child relation has not attracted much attention in existing studies. Furthermore, parent-child relation is a complex concept with static meaning, dynamic meaning, multiple dimensions, and bidirectional features [25], but most related studies only focus on the static meaning and the relation between a certain dimension and children’s resilience, its dynamic meaning, other dimensions, and bidirectional features are neglected, which makes it hard to reflect the true impact of parent-child relation.

This study aims to explore the impact of parental remote migration and parent-child relation on the psychological resilience of left-behind children. First, based on typology, we introduce the concept of parent-child relation types and identify parent-child relation types by latent class analysis. Second, we analyze the impact of parental migration characteristics (spatial and temporal) on the psychological resilience of left-behind children. Third, we examine the impact of father-child relation types, mother-child relation types, and combinations of parent-child relation type on the psychological resilience of left-behind children. This study can provide a theoretical basis and empirical evidence for making targeted intervention programs to improve the psychological resilience of left-behind children.

## 2. Literature Review

### 2.1. Psychological Resilience and Related Theories

Definitions on resilience are divided into three categories. First, some studies define it as an individual’s ability, quality, or trait [11], and everyone has resilience [12]. Second, some studies define it as a positive result of individuals after experiencing adversities [13], or as a mental health status or successful adaptation of individuals in adversities [14]. Third, some studies define it as a dynamic process that individuals actively adapt and develop in adversities [15,16]. In fact, all these definitions reflect the essence of resilience, and should be included in its concept [26]. The concept of resilience can be used to analyze left-behind children in China [15]. 

Based on these definitions and literature [26], this study defines the psychological resilience of left-behind children as a process and result of achieving active adaptation and good development by using various resources and forming comprehensive power. Various resources, resources distributed inside and outside children, are the prerequisite for resilience. Comprehensive power, including personal power and supporting power, is the core element of resilience. Personal power, the power within children, is formed by using various resources and includes three dimensions. Supporting power, the power outside children, includes two dimensions. The sum of personal power and supporting power constitutes the overall level of psychological resilience.

Three theories can be used to analyze the influencing factors of psychological resilience. According to framework of risk and resilience [9,16,27,28,29], psychological resilience is affected by risk and protective factors. Risk factors have negative impacts and lead to adverse consequence, while protective factors can buffer the negative impacts of risk factors and enhance psychological resilience. Since factors inside children are hard to change in a short term, seeking protective factors outside becomes an important strategy to enhance their psychological resilience. Among factors outside, family factors are the most important, and parent-child relation bear the brunt. For the psychological resilience of left-behind children, parental remote migration, and bad parent-child relation may be risk factors, and good parent-child relation may be protective factors.

According to family system theory [30,31], family is a complex system composed of parent subsystem, parent-child subsystem, and sibling subsystem. The change of subsystems and the interaction among subsystems affect children’s development. The change of one subsystem causes the change of others, and the stability and harmony of family system is important to children’s development. The interaction between parents and children forms father-child, mother-child, and parental relation, which affect children’s development. For left-behind children, family system is teared by parental migration, and parent-child relation has become a special kind of relation across the space, which may affect their psychological resilience. 

Ecosystem theory advocates that children’s development be examined in an ecosystem composed of various layers of environment systems [32,33]. It argues that children’s development is the process and result of interaction between individuals and environment systems, and the impact of proximal environment systems is the most direct and powerful. It stresses that family is one of the proximal environment systems in which children are affected throughout lives, and school is also one proximal environment system which affects children’s development. Hence, both family environment composed of parents and school environment composed of teachers and students may affect the psychological resilience of left-behind children.

### 2.2. Population Migration and Psychological Resilience

Studies have shown that family factors (structure, relation, atmosphere, cohesion, and support) are important factors affecting children’s psychological resilience [27,29]. For left-behind children, parental migration will lead to changes in these factors, and then affect their psychological resilience. Studies have also shown that parental migration has significant impacts on the mental health of left-behind children [3], and psychological resilience can guarantee and promote the mental health of left-behind children [20,34]. Therefore, parental migration may weaken left-behind children’s psychological resilience and then threaten their mental health.

It is still rare in existing studies to analyze the influencing factors of left-behind children’s psychological resilience from perspective of parental migration. Only a few studies have explored the relation between psychological resilience and parental migration (mode, duration, and distance), and found that left-behind children have poorer psychological resilience than their peers [35,36,37], those whose mother migrated have poorer psychological resilience than those whose father migrated [35,38], their psychological resilience is negatively correlated with the time of parental migration [35,39] and significantly affected by the time of mother’s migration [40]. Furthermore, it has been shown that the distance of parental migration negatively affects left-behind children’s psychological resilience [41].

### 2.3. Parent-Child Relation and Psychological Resilience

There are different definitions on parent-child relation in different disciplines. Referring to a related study [25], combined with the actual situation, this study defines the parent-child relation of left-behind children as the process of forming emotional attachment between migrant parents and left-behind children through contacting, communicating, interacting, parenting, assisting, etc. Emotional attachment is the dimension of parent-child relation in the static sense, while contacting, communicating, interacting, parenting, and assisting are the dimensions of parent-child relation in the dynamic sense, which are the basis of emotional attachment.

Existing studies analyzing the impact of parent-child relation on the psychological resilience of left-behind children mainly focus on a single dimension of parent-child relation and conclude that parent-child emotional attachment is positively correlated with their psychological resilience, good parent-child emotional attachment can strengthen their psychological resilience. Some studies find that there is gender difference in the association between parent-child emotional attachment and the psychological resilience of left-behind children, where in, mother-child emotional attachment is more correlated with their psychological resilience and has a greater impact their psychological resilience than father-child emotional attachment [42]. 

A few studies have shown that there is significant difference in the psychological resilience of left-behind children who contact or communicate with their parents at different frequencies. The psychological resilience of those who contact parents one time per week is significantly stronger than that of those who contact parents one time per month and those who contact parents one time in three months [43]. It is also shown that there is significant difference in the psychological resilience of those whose parents return home at different frequency, their psychological resilience increases with the increase of parental frequency of returning home [35].

In addition, related studies have proved that parenting style significantly affects left-behind children’s psychological resilience, and there is gender difference in the impact of parenting style [41]. Particularly, the higher the dimension score of parental warmth, the higher the psychological resilience of left-behind children [44], and parental severe punishment is a key factor which limits left-behind children’s psychological resilience [45].

### 2.4. Parent-Child Relation Types and Psychological Resilience

The types of parent-child relation are the results of classifying parent-child relation by some standards and methods. Every type of parent-child relation has its clear meanings and distinct characteristics, and different types of parent-child relation have different characteristics, which essentially reflect the value of classification standards. So, the standards are of great importance for classifying the types of parent-child relation.

However, studies on parent-child relation types are scarce, and their classification standards are not suitable. Some studies chose gender, age, or migration mode as the classification standards, which cannot reflect the essential attributes of parent-child relation. Other studies chose emotional attachment or parenting style as the classification standards, which cannot reflect the multifaceted nature of parent-child relation. Parent-child relation includes multiple dimensions, and they have different functions, different consequences, potential linkages, and they are not simply additive, which makes it hard to be measured only by a certain single dimension.

Fortunately, typology methods (e.g., latent class analysis (LCA)) have more advantages in examining those multidimensional social relationships (e.g., intergenerational relation) [46,47,48]. We argue that LCA can be used to examine the parent-child relation among rural Chinses families. Emotional attachment, communicating, parenting, and assisting are all important dimensions of parent-child relation. Emotional attachment is the dimension of parent-child relation in the static sense, while communicating, parenting, and assisting are the dimensions of parent-child relation in the dynamic sense. The characteristics of these dimensions are exactly the standards when using LCA to identify the types of parent-child relation, which assures the types identified by LCA can reflect the essential attributes, internal structure, and external appearance of parent-child relation better than those classified by other standards and methods.

### 2.5. Limitations of Existing Studies and Innovation of this Study

There are limitations in existing studies, which leaves space for this study. First, some studies have explored the relation between parental migration and left-behind children’s psychological resilience, but the time of parental first migration and the migration of left-behind children are ignored. According to life course theory, the time of parent-child separation will affect children’s later development, and the migration experience of children will affect their current development. Particularly, population migration has different spatial and temporal characteristics, which may have different impacts on left-behind children’s psychological resilience. Therefore, we include the spatial and temporal characteristics of father’s, mother’s, and children’s migration. Second, a few studies have explored the association between parent-child relation and left-behind children’s psychological resilience, but the static dimensions, dynamic dimensions, and bidirectional characteristics of parent-child relation are ignored. Therefore, we considered its multiple dimensions and bidirectional characteristics when defining and measuring parent-child relation, and conducts LCA to identify its latent types. Third, the roles and responsibilities of the father and mother in a family are different, father-child relation and mother-child relation are different, and their impacts on children may be different. Therefore, we analyzed the impacts of parent-child relation types on left-behind children’s psychological resilience from gender perspective of parents so as to reveal the different impacts of father and mother. Fourth, father and mother affect children together, but the common impact of father-child relation and mother-child relation is neglected. In a family, the father-child relation type and mother-child relation type may be the same or not, thus there are various combinations of parent-child relation type between parents. Referring to related study [49], we proposed the concept of combinations of parent-child relation type and analyzes their impacts on left-behind children’s psychological resilience to reflect the common impact of father-child relation and mother-child relation.

## 3. Materials and Methods

### 3.1. Data

The data used in this study came from the China Students Development Survey organized by School of Public Policy and Administration of Xi’an Jiaotong University in Ye county of Henan province in 2015, and in Chenggu county and Ningqiang county of Shaanxi province in 2016.

In recent years, the regional characteristics of population migration in China have been changing. Compared with eastern regions, central and western regions have attracted more migrant workers. More and more migrant workers are transferring from eastern and southern regions to central and western regions. So, it is suitable to select central and western regions for investigation, which can reflect the status and trend of population migration in China.

Henan is a large province with rich migrant workers in central China, and Ye county is a large county of agriculture in Henan. Shaanxi is a large province with rich migrant workers in western China, and Chenggu county and Ningqiang county are subordinate to Hanzhong city in Shaanxi. Especially, there are many left-behind children in the three counties. Therefore, the three counties are ideal areas to study problems of left-behind children.

Stratified Cluster Sampling was used in both surveys. First, 10 junior middle schools were selected in the three counties (4 in Ye county, 3 in Chenggu county, and 3 in Ningqiang county). Second, 2 or 3 classes were selected in each grade from 7 to 9 in selected schools. Finally, all students in selected classes were investigated. Since students were informed before investigation that leave would not be taken for no reason, the proportion of those absent at investigation time was very small. Moreover, the rate of enrolment in surveyed areas was 100%. Therefore, the investigated students were highly representative.

Both surveys were conducted by well-trained class teachers and experienced instructors. First, class teachers distributed questionnaires to students and retracted on the spot after all students finished. Second, class teachers checked questionnaires and gave back to instructors if no problems were found. Finally, instructors checked questionnaires and dealt with problems in time. All distributed questionnaires were returned. Strict quality control methods were adopted in data entry and cleaning. These measures guaranteed the reliability of survey data.

According to the purposes of this study, two groups of left-behind children were selected: (1) whose parents had gone to other cities or towns for work in their local province and (2) whose parents had gone to other cities or towns for work in other provinces.

Table 1 shows the demographic characteristics of the samples.

### 3.2. Variables

#### 3.2.1. Dependent Variable

Psychological resilience was measured by adolescent psychological resilience scale developed on the basis of the process model of resilience and the uniqueness of Chinese children [26]. This scale is widely used and shown to be suitable for Chinese middle school students [41]. It includes 27 items, which are all rated on a five-point scale (completely inconsistent, relatively inconsistent, moderately consistent, relatively consistent, completely consistent), yielding a total score between 27 and 135. The higher the score, the stronger the psychological resilience. In this sample, the Cronbach’s *α* was 0.82, the mean score was 91.62, accounting for 67.87% of total score, which indicated that the overall level of psychological resilience was not optimistic.

#### 3.2.2. Independent Variable

##### Population Migration Characteristics

Spatial characteristics of population migration mainly refer to parents’ and children’s migration distance in the latest 6 months. Therefore, 6 months of parent-child separation was a widely used standard to judge left-behind children [50]. Migrant workers usually return to hometown during the Spring Festival, and invite children to visit them in summer holiday, which means the time span of parent-child separation was about 6 months. Therefore, the migration status of parents and children in the latest 6 months can represent the current migration of population. 

It was difficult for children to supply accurate information on migration distance, so we used working place to measure parents’ migration distance. The respondents were asked to answer the question, “where does your father / mother work?”, based on their answers, we judged their father/mother worked in other cities of local province or other provinces. 

Spatial characteristics of children’s migration were measured by their experience of visiting parents in the latest 6 months. The respondents were asked to answer the question, “have you been to father’s / mother’s working place: (1)Yes, go there to school, (2)Yes, go there to play, and (3) No?” (1) and (2) were defined as “Yes”, and (3) was still defined as “No”.

Temporal characteristics of population migration mainly refer to parents’ and children’s migration experience during left-behind children’s life course. Participants were asked to provide the migration status of parents and themselves at their different ages (1–15). Since students in lower grade(s) were unable to provide information on higher grade(s), according to the classification of children’s key age stages [50] and the key turning points in children’s life course, the period from the birth of a child to the time of survey was divided into three stages: 1–3 years of age (infants), 4–6 years of age (preschool), and 7–10 years of age (primary school), ensuring as much information as possible included in the analysis. If father’s and/or mother’s first migration occurred at a certain age of a child, it was recorded as “yes”, if father’s and/or mother’s first migration did not occur at a certain age of a child, it was recorded as “no”. 

Temporal characteristics of children’s migration were measured by their experience of living or studying in other cities or provinces during their age of 1–10. If they had such experience, it was corded as “yes”; if not, it was corded as “no”.

The characteristics of population migration is detailed in Table 2.

##### Parent-Child Relation Types

Table 3 shows the measurement of parent-child relation dimensions, which are the basis of identifying parent-child relation types. 

According to related study [51] and the requirements of LCA, the options of related variables should be divided into two categories to ensure there are enough samples for each classification in the column list. Because data of parent-child emotion has obvious positive bias characteristics [52], two new variables were generated: “you love parents very much” and “parents care about you very much”; the original option 5 was recoded to 1 = yes, and other original options were recoded to 0 = no. The impact of parent-child separation on children’s mental health may be moderated by more frequent parent-child communication. Therefore, we selected a higher standard when generating new variables of parent-child communication. Two new variables were: “parents contact you frequently” and “you like talking to parents”. The original option 3 was recoded to 1 = yes, and other original options were recoded to 0 = no. With regard to parenting function, the original items and options were still used. New generated variables (in italics) are shown in Table 3. 

Parent-child relation types identified by LCA and combinations of parent-child relation type are shown in “Results” section.

#### 3.2.3. Controlled Variable

According to related studies [35,41], the realities of population migration and the characteristics of left-behind children, five kinds of variables were controlled. 

First, personal characteristics included the gender and age of left-behind children. Second, family characteristics included: Parental Relation was measured by asking how often their parents quarreled, if they never quarreled, it was recorded as good (1), or it was recorded as not good (0); Grandparents Guardianship was measured by asking whether their grandparents lived with them, if any of grandparents lived with them, it was recorded as yes (1), or it was recorded as no (0); Only Child was measured by asking how many siblings they had, if they had no siblings, it was recorded as yes (1), or it was recorded as no (0); and Weekly Cost of Living was measured by asking how much they spent on living per week. Third, school characteristics included: Friends’ Support was measured by friends’ support dimension of Perceived Social Support Scale, it was a continuous variable, the higher the score, the higher the level of support; Students’ Friendliness was measured by asking “are your classmates friendly to you?” the answers included all friendly (5), most friendly (4), half friendly (3), few friendly (2), and all unfriendly (1); and Teacher’s Care was measured by asking “do your teachers care about you?” the answers included all (5), most (4), half (3), few (2), and none (1). Forth, parents can hardly attend the parents’ meeting because of long distance, high transportation costs and busy work, but teachers can do home visits. Family-school interaction was measured by asking “do your teachers do home visits?” the answers include often (2), sometimes (1), and never (0). Fifth, regions included Chenggu county, Ningqiang county, and Ye county.

Characteristics of controlled variables are shown in Table 4.

### 3.3. Statistical Analysis

LCA was used to identify types of parent-child relation, and cross-analysis was used to create combinations of parent-child relation type. According to related studies [46,47,48,53], LCA allows us to capture the association existing among observed categorical indicators by a set of unobserved latent classes; latent class probabilities and conditional probabilities are the two parameters estimated in LCA, latent class probabilities describe the distribution of classes of the latent variable, while conditional probabilities represent the degree of association between each observed variable and each latent class. 

In identifying types of parent-child relation, we started by computing a latent class model with only a single latent class and added one class after another, checking for model fit and significance. Then, based on goodness-of-fit indicators (e.g., likelihood ratio chi-square (L^2^), akaike information criterion (AIC), bayesian informal criterion (BIC)) of latent class models, we selected the “best” model and determined the number of latent classes (types). Finally, based on latent class probabilities and conditional probabilities of the “best” model, we assigned the labels (names) to describe the latent classes identified in the “best” model. 

Correlation analysis was used to check the correlation between parent-child relation types and the psychological resilience of left-behind children. Then, hierarchical linear regression analysis was performed to analyze the influencing factors associated with the psychological resilience of left-behind children.

The strategies of regression analysis were: Model 1 analyzed the impacts of characteristics of children, family, school, home-school interaction, and region. Based on Model 1, migration characteristics of father, mother, and children were added in Model 2 to reveal the impacts of population migration. Based on Model 2, father-child relation types were added in Model 3 to reveal the independent impacts of father-child relation types. Based on Model 2, mother-child relation types were added in Model 4 to reveal the independent impacts of mother-child relation types. Based on Model 2, both father-child relation types and mother-child relation types were added in Model 5 to compare their different impacts. Based on Model 2, combinations of parent-child relation type were added in Model 6 to analyze the common impact of father and mother.

The software Mplus (version 7.4; Muthén & Muthén, Los Angeles, CA, USA) was used for LCA. The software SPSS (version 22; SPSS Inc., Chicago, IL, USA) was used for statistical analysis. 

## 4. Results

### 4.1. Parent-Child Relation Types and Combinations of Parent-child Relation Type

#### 4.1.1. Parent-Child Relation Types

The goodness-of-fit statistics of six latent class models are summarized in Table 5. Among goodness-of-fit indicators, the BIC is widely used when selecting the best-fitting model among competing models, the smaller the BIC, the better the model fit [46,47,48,53]. Table 5 shows successive decreases in the size of the BIC with each additional latent class added, indicating relative improvements in fit, up to five classes. The BIC then increased in the six-class model, indicating the superiority of the five-class model. Therefore, five-class model is the “best” model.

Latent class coefficients (latent class probabilities and conditional probabilities) for five-class model of parent-child relation (Table 6) are the basis for naming the latent classes (types).

Compared with type 2–5, children belonging to type 1 have the deepest emotional attachment and the most frequent communication, which indicates that they have intimate connection with parents. Meanwhile, parents belonging to type 1 have the strongest parenting function. Thus, type 1 can be named “intimate connection and strong function”. 

Compared with type 3–5, children belonging to type 2 have deeper emotional attachment and more frequent communication, which indicates that they have close connection with parents. Meanwhile, parents belonging to type 2 have the weakest parenting function. Thus, type 2 can be named “close connection but lacking function”. 

The characteristics of coefficients in type 3 are quite distinct. Among 5 types, the coefficients degrees of three dimensions are neither high nor low. Thus, type 3 can be named “moderate connection and function”. 

Compared with type 1–3, both emotional attachment and communication quality of type 4 is relatively weak. Compared with type 5, parenting function of type 4 is stronger. Thus, type 4 can be named “estranged connection but strong function”. 

Compared with type 1–4, emotional attachment of type 5 is the weakest, communication quality is very low, and parenting function is very weak. Thus, type 5 can be named “alienation connection and weak function”.

Table 7 provides characteristics of father-child relation types and mother-child relation types. Father-child relation of “close connection but lacking function” takes the highest proportion (33.02%), father-child relation of “alienation connection and weak function” takes the second (28.75%), and father-child relation of “estranged connection but strong function” takes the lowest (7.9%). Mother-child relation of “close connection but lacking function” takes the highest proportion (28.21%), mother-child relation of “intimate connection and strong function” takes the second (27.26%), and mother-child relation of “moderate connection and function” takes the lowest (10.30%). It is seen that father-child relation types and mother-child relation types in Chinese left-behind families are obviously different.

#### 4.1.2. Combinations of Parent-Child Relation Type

The 25 combinations of parent-child relation type were formed by cross-analyzing father-child relation types and mother-child relation types. To ensure the regression analysis was feasible and the results were credible, combinations of small proportion and poor representation were removed, and combinations accounting for 5% and above were remained as shown in Table 8. 

### 4.2. Correlation of Parent-Child Relation Types and Psychological Resilience

Table 9 shows the correlation analysis results of parent-child relation types and left-behind children’s psychological resilience. 

There is significantly positive correlation between psychological resilience and parent-child relation of FCRT1, MCRT1, FCRT2, and MCRT2. There is significantly negative correlation between psychological resilience and parent-child relation of FCRT5, MCRT4, and MCRT5. There is no significant correlation between psychological resilience and FCRT3, FCRT4, and MCRT3. It is inferred that FCRT1, FCRT2, MCRT1, and MCRT2 may be conducive to improve left-behind children’s psychological resilience, while FCRT5 and MCRT5 may not. 

### 4.3. Impact of Population Migration and Parent-Child Relation Types

#### 4.3.1. Impact of Controlled Variables

It is seen from model 1 (Table 10) that good relation between parents has a significantly positive impact on left-behind children’s psychological resilience, especially for those long-distance migrating parents; among left-behind children, the only child’s psychological resilience is obviously stronger; friends’ support, teachers’ care, and classmates’ friendliness have significantly positive impacts, the more supportive the friends are, the more care the teachers give, and the more friendly the students are, the stronger left-behind children’s psychological resilience is; home-school interaction has a significant impact, compared with teachers who never do home visits, teachers who sometimes do home visits can significantly improve left-behind children’s psychological resilience, but teachers who often do home visits cannot; gender, age, weekly cost of living, grandparent’s guardianship, and living in school have no significant impacts on left-behind children’s psychological resilience.

#### 4.3.2. Impact of Population Migration

It is seen from model 2 (Table 10) that different characteristics of population migration have different impacts on left-behind children’s psychological resilience. Both spatial and temporal characteristics of father’s migration have no significant impacts. The spatial characteristics of mother’s migration have significant impacts. Compared with left-behind children whose mothers work in the local province, those whose mothers work in other provinces have poorer psychological resilience. The temporal characteristics of mother’s migration have no significant impacts, and left-behind children’s migration characteristics have no significant impacts, either.

#### 4.3.3. Impact of Father-Child Relation Types

It is seen from model 3 (Table 10) that after various characteristics variables are controlled, father-child relation types have significant impact on left-behind children’s psychological resilience. Compared with father-child relation of “alienation connection and weak function” (FCRT5), father-child relation of “close connection but lacking function” (FCRT2), “estranged connection but strong function” (FCRT4), “intimate connection and strong function” (FCRT1), and “moderate connection and function” (FCRT3) have significantly positive impacts on left-behind children’s psychological resilience, and their impacts were from big to small.

#### 4.3.4. Impact of Mother-Child Relation Types

From the results of model 4 (Table 10), it can be seen that mother-child relation types have significant impacts on left-behind children’s psychological resilience. Compared with mother-child relation of “alienation connection and weak function” (MCRT5), only mother-child relation of “intimate connection and strong function” (MCRT1) has significantly positive impact on left-behind children’s psychological resilience.

The results of model 4 and model 3 show that the impacts of father-child relation types and mother-child relation types on left-behind children’s psychological resilience are similar, both FCRT1 and MCRT1 have significantly positive impacts. Meanwhile, the impact of father-child relation types is also different from that of mother-child relation types, all types of father-child relation significantly affect left-behind children’s psychological resilience, but only MCRT1 affects. 

#### 4.3.5. Difference in Impact of Parent-Child Relation Types

The results of model 5 (Table 10) show that only father-child relation types have significant impact on the psychological resilience of left-behind children. Compared with FCRT5, FCRT4 has significantly positive impact on the psychological resilience of left-behind children, while other three types of father-child relation have no significant impact.

#### 4.3.6. Impact of Combinations of Parent-Child Relation Type

The results of model 6 (Table 10) show that compared with combination of “parental alienation connection and weak function” (FCRT5-MCRT5), combination of “paternal close connection but lacking function-maternal intimate connection and strong function” (FCRT2-MCRT1), and combination of “parental intimate connection and strong function” (FCRT1-MCRT1) have positive impacts on left-behind children’s psychological resilience, and their impacts are from large to small, while other four combinations of parent-child relation type have no significant impacts.

## 5. Discussion

It has been confirmed that parental migration affects the psychological resilience of left-behind children [35,36,37,38,39,40,41], which is echoed by the finding of this study that different characteristics of population migration have different impacts on left-behind children’s psychological resilience. Specifically, the spatial characteristics of mother’s migration have significant impact on left-behind children’s psychological resilience, compared with left-behind children whose mothers work in local province, those whose mothers work in other provinces have poorer psychological resilience. This finding is similar to the finding of our previous study that the distance of parental migration negatively affects the psychological resilience of left-behind children [41], which, thus, complements the findings of others and suggests parents to avoid long-distance migration. 

At the same time, it is shown that the spatial characteristics of father’s migration have no significant impact on left-behind children’s psychological resilience, which indicates that father’s absence from home has no impact on children’s psychological resilience, but mother’s absence does. Existing studies have also explored that left-behind children whose father migrated have stronger psychological resilience than those whose mother migrated [35,38]. One reason for the difference in the impact of parental migration is that they take different duties and play different roles in a family, which has been discussed in numerous studies. In rural Chinese families, mothers, as the main caregivers, are usually closer to children than fathers, which means that mother’s migration may have more negative impacts than fathers. Therefore, compared with mother migration, father migration may be a better decision when one parent has to migrate.

In addition, a few studies have found the negative correlation between the time of parental migration and the psychological resilience of left-behind children [35,39,40], which is enriched by the finding of this study that the time of parental first migration has no significant effect on the psychological resilience of left-behind children. This study also examined the impact of left-behind children’s migration characteristics, and found it was not significant. But this was a useful attempt, which reminds researchers that the migration experience left-behind children may have should not be neglected.

The finding that father-child relation types have significant impacts on left-behind children’s psychological resilience may indicate that types of relation between migrant fathers and left-behind children are important for left-behind children’s psychological resilience, which was rarely emphasized in previous studies. Compared with FCRT5, other four types of father-child relation have significantly positive impacts, and FCRT2 affects the most, which indicates that FCRT5 is the worst and risk factor for left-behind children’s psychological resilience, while FCRT2 is the best and the “umbrella”. This finding suggests that migrant fathers should strive to transform their types of relation with their children into FCRT2. In addition, father’s migration has no significant impact on left-behind children’s psychological resilience, which may be due to the compensation effect of father-child relation types.

It turned out that mother-child relation types also have significant impacts on left-behind children’s psychological resilience, which is similar to father-child relation types. Specifically, compared with MCRT5, only MCRT1 has significantly positive impact, which is different from father-child relation types. This finding also indicates that MCRT5 is the worst and risk factor for left-behind children’s psychological resilience, while MCRT1 is the best and the “umbrella”. Therefore, migrant mothers should try to change their types of relation with children into MCRT1. The finding that father-child relation types have more influence on left-behind children’s psychological resilience than mother-child relation types is also compelling, which contrasts with the results of related studies that mother-child emotional attachment has more influence on left-behind children’s psychological resilience than father-child emotional attachment [42].

It is true that there are different combinations of father-child relation type and mother-child relation type in different families, and they may have different impacts on children’s psychological resilience. Compared with combination of FCRT5-MCRT5, combinations of FCRT2-MCRT1 and FCRT1-MCRT1 have significant impacts on left-behind children’s psychological resilience, which indicates that combination of the best father-child relation type and the best mother-child relation type is the best and protective factor for left-behind children’s psychological resilience, while combination of the worst father-child relation type and the worst mother-child relation type is the worst and risk factor. This finding is similar to the finding of our related study that combination of positive parenting style between father and mother has significantly positive impact on migrant children’s life satisfaction, while combination of negative parenting style between father and mother has significantly negative impact [48]. Our findings together with others [54,55] indicate that children’s mental health and behaviors are commonly influenced by father and mother, which suggests that migrant parents should recognize and transfer to the best combination of parent-child relation type or parenting style so as to guarantee and improve their children’s mental health.

Last but not least, the findings of this study have practical implications for policymakers in China. More attention should be paid to the improvement of left-behind children’s psychological resilience. First, local governments should promote both new urbanization strategy and rural revitalization strategy to change the migration mode of rural labor from “remote” to “local”. Second, rural parents should optimize the family migration decision and try to avoid remote migration and mother migration. Third, both father and mother should try to shape a better type of relation with children and a better type of each other. Fourth, schools, especially teachers, should assume more responsibility and highlight moderate home-school interaction to compensate the loss of parental absence. 

Limitations need to be acknowledged. First, the survey was conducted in central and western regions, which may limit the representativeness of samples. New survey data collected in eastern regions is to be used in future studies. Second, the impact of father’s and mother’s migration on left-behind children’s psychological resilience may differs between boy(s) and girl(s), whether the mechanisms are same or not is interesting but not answered, which should be concerned in future studies. Third, parental migration may affect parent-child relation, and then affect left-behind children’s psychological resilience, which is a valuable mechanism worthy tested in future studies. Last, parental migration and changes in parent-child relation may affect teacher-student relation and peer relation, then affect left-behind children’s psychological resilience, which should be checked in future studies.

## 6. Conclusions

This study mainly analyzed the impact of parental remote migration and parent-child relation types on the psychological resilience of Chinese rural left-behind children. In the context of massive population rural-urban migration, mother’s remote migration did exert negative impacts on the psychological resilience of left-behind children, while father’s remote migration did not. In Chinese rural families, different father-child relation types, mother-child relation types, and combinations of parent-child relation type had different impacts on left-behind children’s psychological resilience. Teachers and friends played important roles in the psychological resilience of left-behind children, their relations with left-behind children, father-child relation, mother-child relation, and parental relation together constituted an external network that affects left-behind children’s psychological resilience. Special attention ought to be paid to this external network and parental migration. More targeted policies are encouraged to promote the psychological resilience of left-behind children.

## Figures and Tables

**Table 1:** Demographic characteristics of the samples.

Variables	Cases (*n*)	Frequency (%)
**Sex**		
Female	311	48.10
Male	335	51.90
**Grade**		
7	213	33.00
8	231	35.80
9	202	31.30
**Only Child** (dushengzinv)		
No	481	74.50
Yes	165	25.50
**Living in School**		
No	253	39.20
Yes	393	60.80
**Region**		
Ningqiang county	197	30.50
Chenggu county	146	22.60
Ye county	303	46.90

**Table 2:** Characteristics of population migration.

Variables	Description	*n*	%
**Spatial Characteristics of Father’s Migration**			
Working place	0 = other cities of local province	118	18.27
	1 = other province	528	81.73
**Temporal Characteristics of Father’s Migration**			
First migration occurred when child aged 1–3	0 = no	218	40.30
	1 = yes	323	59.70
First migration occurred when child aged 4–6	0 = no	181	30.63
	1 = yes	410	69.37
First migration occurred when child aged 7–10	0 = no	132	21.96
	1 = yes	469	78.04
**Spatial Characteristics of Mother’s Migration**			
Working place	0 = other cities of local province	128	19.81
	1 = other province	518	80.19
**Temporal Characteristics of Mother’s Migration**			
First migration occurred when child aged 1–3	0 = no	291	58.79
	1 = yes	204	41.21
First migration occurred when child aged 4–6	0 = no	219	42.03
	1 = yes	302	57.97
First migration occurred when child aged 7–10	0 = no	171	30.70
	1 = yes	386	69.30
**Spatial Characteristics of Child’s Migration**			
Has been to father’s working place	0 = no	303	46.90
	1 = yes	343	53.10
Has been to mother’s working place	0 = no	248	42.32
	1 = yes	338	57.68
**Temporal Characteristics of Child’s Migration**			
Living in other cities or provinces	0 = no	439	67.96
	1 = yes	207	32.04

**Table 3:** Measurement of parent-child relation dimensions.

Variables	Description
**Parent-Child Emotional Attachment**	
How much do you love parents	1 = not at all; 2 = not quite; 3 = moderate; 4 = quite; 5 = very
How much do parents care about you	1 = not at all; 2 = not quite; 3 = moderate; 4 = quite; 5 = very
*You love parents very much*	*0 = no; 1 = yes*
*Parents care about you very much*	*0 = no; 1 = yes*
**Parent-Child Communication**	
How often parents contact you	1 = never; 2 = sometimes; 3 = frequently
How do you like talking to parents	1 = dislike; 2 = moderate; 3 = like
*Parents contact you frequently*	*0 = no; 1 = yes*
*You like talking to parents*	*0 = no; 1 = yes*
**Parenting Function**	
Supervise and urge you study most frequently	0 = no; 1 = yes
Help and support you the most	0 = no; 1 = yes

**Table 4:** Characteristics of controlled variables.

Variables	Definition	*n*	%	Mean	*S.D.*
Sex	0 = female	311	48.14		
	1 = male	335	51.86		
Age	[7,16]	645		13.44	1.112
Parental Relationship	0 = not good	469	75.89		
	1 = good	149	24.11		
Grandparents Guardianship	0 = no	124	20.95		
	1 = yes	468	79.05		
Only Child	0 = no	481	74.46		
	1 = yes	165	25.54		
Weekly Cost of Living	0 = no	33	5.14		
	1 = yes	609	94.86		
Friends’ Support	[4,28]	629		18.86	5.280
Students’ Friendliness	[1,2,3,4,5]	642		4.11	0.749
Teachers’ Care	[1,2,3,4,5]	643		4.15	0.997
Boarding School	0 = no	253	39.16		
	1 = yes	393	60.84		
Teachers’ Home Visit	0 = never	33	5.14		
	1 = sometimes	125	19.47		
	2 = frequently	484	75.39		
Regions	0 = Chenggu county	146	22.60		
	1 = Ningqiang county	197	30.50		
	2 = Ye county	303	46.90		

**Table 5:** Model fit for the optimal number of classes in the latent class analysis (LCA) of parent-child relation.

No. of Classes	Log L.	AIC	BIC	DF	*p*-Value
1	−30,312.676	60,637.351	60,679.041	57	0.0000
2	−28,031.304	56,088.608	56,178.937	50	0.0000
3	−27,784.182	55,608.365	55,747.331	43	0.0000
4	−27,621.462	55,296.925	55,484.530	36	0.0000
5	−27,549.280	55,166.560	55,402.803	29	0.0000
6	−27,520.286	55,122.572	55,407.453	22	0.0000

Log L., log likelihood; AIC, akaike information criterion; BIC, bayesian informal criterion; DF, degree of freedom.

**Table 6:** Latent class coefficients for five-class model of parent-child relations.

Dimensions and Indicators	Type 1	Type 2	Type 3	Type 4	Type 5
**Emotional Attachment**					
You love parents very much	0.916 ***	0.877 ***	1.000	0.238 ***	0.175 ***
Parents care about you very much	0.946 ***	0.862 ***	0.825 ***	0.440 ***	0.162 ***
**Communication Quality**					
Parents contact you frequently	0.839 ***	0.603 ***	0.078	0.336 ***	0.110 ***
You like talking to parents	0.816 ***	0.773 ***	0.368 ***	0.196 ***	0.102 ***
**Parenting Function**					
Supervise and urge you study most frequently	0.654 ***	0.000	0.381 ***	0.583 ***	0.142 ***
Help and support you the most	0.604 ***	0.082	0.276 ***	0.527 ***	0.027

***, *p <* 0.001.

**Table 7:** Characteristics of parent-child relation types.

Variables	*n*	%
**Father-Child Relation Types**		
Intimate Connection and Strong Function (FCRT1)	100	15.80
Close Connection but Lacking Function (FCRT2)	209	33.02
Moderate Connection and Function (FCRT3)	92	14.53
Estranged Connection but Strong Function (FCRT4)	50	7.90
Alienation Connection and Weak Function (FCRT5)	182	28.75
**Mother-Child Relation Types**		
Intimate Connection and Strong Function (MCRT1)	172	27.26
Close Connection but Lacking Function (MCRT2)	178	28.21
Moderate Connection and Function (MCRT3)	65	10.30
Estranged Connection but Strong Function (MCRT4)	72	11.41
Alienation Connection and Weak Function (MCRT5)	144	22.82

**Table 8:** Combinations of parent-child relation type between father and mother (*n =* *494*).

Combinations	*n*	%
FCRT1-MCRT1	35	7.45
FCRT1-MCRT2	53	11.28
FCRT2-MCRT1	94	20.00
FCRT3-MCRT3	42	8.94
FCRT2-MCRT2	90	19.15
FCRT5-MCRT4	50	10.64
FCRT5-MCRT5	106	22.55

FCRT1-MCRT1, “parental alienation connection and weak function”; FCRT1-MCRT2, “parental close connection but lacking function”; FCRT2-MCRT1, “paternal close connection but lacking function - maternal intimate connection and strong function”; FCRT3-MCRT3, ”parental moderate connection and function”; FCRT2-MCRT2, “parental close connection but lacking function”; FCRT5-MCRT4, “paternal alienation connection and weak function - maternal estranged connection but strong function”; FCRT5-MCRT5, “parental alienation connection and weak function”.

**Table 9:** Correlation of parent-child relation types and psychological resilience.

	Father-Child Relation Types					Mother-Child Relation Types				
Item	FCRT1	FCRT2	FCRT3	FCRT4	FCRT5	MCRT1	MCRT2	MCRT3	MCRT4	MCRT5
PP	0.102 *	0.083 *	0.003	0.001	−0.170 **	0.127 **	0.035	−0.007	−0.050	−0.130 **
SP	0.157 **	0.116 **	0.039	−0.018	−0.269 **	0.172 **	0.121 **	0.004	−0.108 **	−0.236 **
PR	0.138 **	0.121 **	0.026	−0.005	−0.254 **	0.165 **	0.100 *	0.002	−0.090 *	−0.217 **

PP, personal power; SP, supporting power; PR, psychological resilience; ** *p* < 0.01; and * *p* < 0.05; Intimate Connection and Strong Function (FCRT1); Close Connection but Lacking Function (FCRT2); Moderate Connection and Function (FCRT3); Estranged Connection but Strong Function (FCRT4); Alienation Connection and Weak Function (FCRT5); Intimate Connection and Strong Function (MCRT1); Close Connection but Lacking Function (MCRT2); Moderate Connection and Function (MCRT3); Estranged Connection but Strong Function (MCRT4); Alienation Connection and Weak Function (MCRT5 ).

**Table 10:** Impacts of population migration characteristics and parent-child relation types.

Variables	Regression Coefficient (Significance)					
	Model 1 [*Beta*]	Model 2 [*Beta*]	Model 3 [*Beta*]	Model 4 [*Beta*]	Model 5 [*Beta*]	Model 6 [*Beta*]
**Personal Characteristics**						
Sex (Female)	−0.061	−0.057	−0.063	−0.035	−0.058	−0.051
Age	−0.064	−0.113 *	−0.115 *	−0.105 *	−0.115 *	−0.083
Region (Ningqiang county)						
Chenggu county	−0.004	0.050	0.079	0.055	0.091	0.080
Ye county	0.057	0.077	0.100	0.070	0.111	0.085
**Family Characteristics**						
Parental Relation (Not Good)	0.094 *	0.104 *	0.097 +	0.088 +	0.092 +	0.106 +
Grandparents Guardianship (No)	−0.061	−0.056	−0.064	−0.055	−0.059	−0.083
Only Child (No)	0.085 *	0.098 +	0.095 +	0.085	0.091 +	0.138 *
Weekly Cost of Living (No)	0.004	0.022	0.017	0.024	0.018	0.020
**School Characteristics**						
Friends’ Support	0.302 ***	0.346 ***	0.343 ***	0.359 ***	0.350 ***	0.364 ***
Students’ Friendship	0.144 ***	0.099 +	0.073	0.074	0.065	0.069
Teachers’ Care	0.148 ***	0.155 **	0.129 *	0.117 *	0.125 *	0.140 *
Boarding School (No)	−0.010	0.009	0.007	0.011	0.000	0.022
**Family-school Interaction**						
Teachers’ Home Visit (Never)						
Sometimes	0.109 **	0.084	0.098 +	0.078	0.094 +	0.064
Frequently	−0.039	0.009	0.009	0.009	0.015	−0.003
**Spatial Characteristics of Father’s Migration**						
Working Place (Other Cities of Local Province)						
Other Province		0.032	0.035	0.024	0.039	−0.061
**Temporal Characteristics of Father’s Migration**						
First-time migration occurred						
When child aged 1–3 (No)		0.070	0.052	0.057	0.054	0.052
When child aged 4–6 (No)		−0.054	−0.042	−0.060	−0.054	−0.147
When child aged 7–10 (No)		0.071	0.041	0.069	0.037	0.106
**Spatial Characteristics of Mother’s Migration**						
Working Place (Other Cities of Local Province)						
Other Province		−0.143 +	−0.149 +	−0.132 +	−0.150 +	−0.054
**Temporal Characteristics of Mother’s Migration**						
First-time migration occurred						
When child aged 1–3 (No)		−0.034	−0.021	−0.014	−0.019	−0.059
When child aged 4–6 (No)		−0.021	0.013	0.005	0.025	0.070
When child aged 7–10 (No)		−0.010	−0.004	−0.002	0.005	0.013
**Spatial Characteristics of Child’s Migration in the Latest 6 Months**						
Has been to father’s working place (No)		0.001	−0.035	−0.017	−0.025	−0.105
Has been to mother’s working place (No)		−0.004	0.006	0.000	−0.002	0.060
**Temporal Characteristics of Child’s Migration 6 Months ago**						
Lived in other cities or provinces (No)		0.016	0.041	0.037	0.047	0.036
**Father-Child Relation Types** (FCRT5)						
FCRT1			0.134 *		0.092	
FCRT2			0.213 ***		0.143	
FCRT3			0.116 *		0.085	
FCRT4			0.163 **		0.171 **	
**Mother-Child Relation Types** (MCRT5)						
MCRT1				0.183 **	0.133	
MCRT2				0.097	0.037	
MCRT3				0.066	0.034	
MCRT4				−0.004	−0.018	
**Combinations of Parent-child Relation Type** (**FCRT5-MCRT5**)						
FCRT1-MCRT1						0.127 *
FCRT1-MCRT2						0.084
FCRT2-MCRT1						0.203 **
FCRT3-MCRT3						0.039
FCRT2-MCRT2						0.082
FCRT5-MCRT4						−0.039
**R^2^**	0.237	0.282	0.311	0.298	0.321	0.348
**Adj. R^2^**	0.215	0.224	0.244	0.229	0.244	0.254
**F**	11.044 ***	4.827 ***	4.600 ***	4.322 ***	4.168 ***	3.691 ***
***n***	512	332	324	324	324	245

Contrast object in small brackets; ***, *p* < 0.001; **, *p* < 0.01; *, *p* < 0.05; and +, *p* < 0.1; Intimate Connection and Strong Function (FCRT1); Close Connection but Lacking Function (FCRT2); Moderate Connection and Function (FCRT3); Estranged Connection but Strong Function (FCRT4); Alienation Connection and Weak Function (FCRT5); Intimate Connection and Strong Function (MCRT1); Close Connection but Lacking Function (MCRT2); Moderate Connection and Function (MCRT3); Estranged Connection but Strong Function (MCRT4); Alienation Connection and Weak Function (MCRT5 ).
